# Sound Segregation via Embedded Repetition Is Robust to Inattention

**DOI:** 10.1037/xhp0000147

**Published:** 2015-10-19

**Authors:** Keiko Masutomi, Nicolas Barascud, Makio Kashino, Josh H. McDermott, Maria Chait

**Affiliations:** 1 Interdisciplinary Graduate School of Science and Engineering, Tokyo Institute of Technology; 2 Ear Institute, University College London; 3 Interdisciplinary Graduate School of Science and Engineering, Tokyo Institute of Technology, and Human Information Science Laboratory, NTT Communication Science Laboratories, NTT Corporation, Kanagawa, Japan; 4 Department of Brain and Cognitive Sciences, Massachusetts Institute of Technology; 5 Ear Institute, University College London

**Keywords:** auditory scene analysis, attention, segregation, streaming, load

## Abstract

The segregation of sound sources from the mixture of sounds that enters the ear is a core capacity of human hearing, but the extent to which this process is dependent on attention remains unclear. This study investigated the effect of attention on the ability to segregate sounds via repetition. We utilized a dual task design in which stimuli to be segregated were presented along with stimuli for a “decoy” task that required continuous monitoring. The task to assess segregation presented a target sound 10 times in a row, each time concurrent with a different distractor sound. McDermott, Wrobleski, and Oxenham (2011) demonstrated that repetition causes the target sound to be segregated from the distractors. Segregation was queried by asking listeners whether a subsequent probe sound was identical to the target. A control task presented similar stimuli but probed discrimination without engaging segregation processes. We present results from 3 different decoy tasks: a visual multiple object tracking task, a rapid serial visual presentation (RSVP) digit encoding task, and a demanding auditory monitoring task. Load was manipulated by using high- and low-demand versions of each decoy task. The data provide converging evidence of a small effect of attention that is nonspecific, in that it affected the segregation and control tasks to a similar extent. In all cases, segregation performance remained high despite the presence of a concurrent, objectively demanding decoy task. The results suggest that repetition-based segregation is robust to inattention.

Human listeners are adept at inferring information about the world from sound, and in doing so solve a number of difficult computational problems. However, most of what we know about the underlying processes is derived from experiments in which listeners are directing attention toward an auditory stimulus. A fundamental question in auditory cognitive neuroscience is to what extent these processes also occur when listeners are not directly attending to sound, as is often the case in everyday life. Must listeners be actively listening to the acoustic input for auditory perceptual organization to occur, or does this transpire automatically irrespective of the focus of attention? Because of its status as the most distal sense, the auditory system is often hypothesized to serve as the brain’s “early warning” system—with one function being to scan the environment for behaviorally relevant events (e.g., Demany, Semal, Cazalets, & Pressnitzer, 2010; Eramudugolla, Irvine, McAnally, Martin, & Mattingley, 2005; Murphy, Fraenkel, & Dalton, 2013). For this reason, one might arguably expect auditory scene analysis to be at least partly automatic and independent of attentional focus. However, experimental results remain equivocal.

The susceptibility of basic auditory processes to attentional load has been mostly studied in the context of brain imaging experiments where brain responses to ignored auditory stimuli are examined while participants engage in a competing task. Results usually reveal decreased activation in auditory areas when listeners direct attention to another modality (e.g., Alain & Woods, 1994; Bidet-Caulet et al., 2007; Ghatan, Hsieh, Petersson, Stone-Elander, & Ingvar, 1998; Hillyard, Hink, Schwent, & Picton, 1973; Johnson & Zatorre, 2005; Kastner & Ungerleider, 2000; Näätanen, 1992; Ross, Hillyard, & Picton, 2010; Snyder, Alain, & Picton, 2006; Teder-Sälejärvi, Hillyard, Röder, & Neville, 1999), which is interpreted as evidence for selective inhibitory modulation of nonrelevant sensory input. However, it remains unclear whether this decreased activation has consequences for auditory perceptual representation and behavior.

For instance, accumulating work shows that the passively elicited mismatch negativity (MMN), a change-evoked brain response generated by infrequent “deviant” events embedded in a stream of repeating standard events (Näätänen, 1992), is not affected by concurrent attentional load (e.g., Alho, Woods, Algazi, & Näätänen, 1992; Bendixen & Schröger, 2008; Chait, Ruff, Griffiths, & McAlpine, 2012; Dyson, Alain, & He, 2005; Muller-Gass, Macdonald, Schröger, Sculthorpe, & Campbell, 2007; Muller-Gass, Stelmack, & Campbell, 2006; Näätänen, Paavilainen, Rinne, & Alho, 2007; Restuccia, Della Marca, Marra, Rubino, & Valeriani, 2005; SanMiguel, Corral, & Escera, 2008; Sculthorpe, Collin, & Campbell, 2008; Sussman, 2007; Sussman, Winkler, & Wang, 2003; Woods, Alho, & Algazi, 1992; but see Alain & Izenberg, 2003; Haroush, Hochstein, & Deouell, 2010; Spielmann, Schröger, Kotz, Pechmann, & Bendixen, 2013; Woldorff & Hillyard, 1991; Zhang, Chen, Yuan, Zhang, & He, 2006). These findings have led to the commonly held view that the mechanisms responsible for the detection of oddball events (events that differ from the preceding context on some acoustic dimension) in the auditory scene are generally independent of attention.

The role of attention in auditory segregation—the recovery of individual sound sources from the aggregate auditory input—remains controversial (Ding & Simon, 2012; Macken, Tremblay, Houghton, Nicholls, & Jones, 2003; Shamma, Elhilali, & Micheyl, 2011; Snyder, Gregg, Weintraub, & Alain, 2012). There is some evidence that segregation based on temporally local cues (such as harmonicity) is largely automatic. For example, the “object-related negativity” (ORN), an event-related brain response, thought to index concurrent sound segregation based on inharmonicity and onset asynchrony, is not affected by attentional load (Alain & Izenberg, 2003; Dyson et al., 2005; Lipp, Kitterick, Summerfield, Bailey, & Paul-Jordanov, 2010). On the other hand, segregation can in some cases be effortfully driven by attentional selection processes that track a dynamic sound source amid competing distractors (Woods & McDermott, 2015). But in many instances, the situation is less clear (Brochard, Drake, Botte, & McAdams, 1999; Carlyon, Cusack, Foxton, & Robertson, 2001; Cusack, Deeks, Aikman, & Carlyon, 2004; Macken et al., 2003; Shamma et al., 2011; Thompson, Carlyon, & Cusack, 2011; Weintraub, Metzger, & Snyder, 2014).

The most studied stimulus in this context has been that of the “A-B-A” streaming paradigm, consisting of a sequence of two tones of different frequencies, A and B, organized into repeating ABA triplets (Bregman, 1994; van Noorden, 1975). For certain stimulus parameters, a segregated percept of two concurrent streams “builds up” after a period of a few seconds. Several attempts to determine whether streaming of this sort depends on attention have used indirect measures of streaming. One approach has measured the effect of task-irrelevant background sounds on a visual recall task (the “irrelevant sound effect”). Such studies have consistently demonstrated that the disruption caused by background sounds is modulated by their perceptual organization into streams, suggestive of preattentive auditory streaming (for review, see Macken et al., 2003). Other studies have drawn similar conclusions from neural measures that could plausibly reflect streaming (Gutschalk, Rupp, & Dykstra, 2015). In contrast to these findings, Carlyon and colleagues found that the build-up of the streaming percept is impaired when attention is shifted away from the streaming signal (Carlyon et al., 2001; Carlyon, Plack, Fantini, & Cusack, 2003; Thompson et al., 2011; see also Alain & Izenberg, 2003). One explanation of their results is that inattention disrupts the processes underlying the segregation of the A and B streams. However, another explanation is that streaming proceeds normally under inattention but is then “reset” when attention is switched to the streaming stimulus (necessary in order to report the streaming percept). This ambiguity underlines the challenge of addressing the effects of attention on streaming using A-B-A paradigms—it is difficult to assess perceptual organization without asking the listener to direct their attention to the stimulus in question.

In this article, we investigate the effects of inattention on streaming using an alternative paradigm that lends itself to probing streaming/segregation when listeners’ attention is diverted away from sound. In prior work, McDermott et al. (2011) demonstrated that listeners can extract a repeating sound source from a dynamically varying background even when other segregation cues are not available. They generated artificial signals that shared coarse statistical properties of natural sounds (spectro-temporal modulation) but that lacked the fine-grained structure believed to underlie most generic grouping cues (e.g., common onset and harmonicity). When presented with sequences of mixtures, listeners were able to recover a repeating novel “target” sound if it was presented in the context of a changing background, as measured by their ability to compare a subsequent probe sound to the target sound. In contrast, when presented with a single such mixture, the same task was impossible. These findings indicate that repetition alone can drive sound segregation (see also Andreou, Kashino, & Chait, 2011; Bendixen, 2014). The mechanism supporting the detection and estimation of the repeating spectro-temporal structure must involve some short-term cache to store the input over the repetition time, and a process (perhaps a form of cross correlation; McDermott et al., 2011) by which this information is compared with the incoming acoustic signal. Preliminary work by McDermott et al. (2011) suggested this temporal integration process extends up to at least 500 ms.

The present article investigates the extent to which repetition-based segregation depends on attention. Many behaviorally relevant sound sources (locomotion sounds, animal calls, etc.) consist of a repeated pattern, lending plausibility to the notion that the auditory system could possess automatic mechanisms to make use of this property for scene analysis (see also Agus, Thorpe, & Pressnitzer, 2010). Notably, the embedded repetition phenomenon lends itself to attentional manipulations, because segregation is assessed after the fact, by asking listeners whether a subsequent probe sound was present in the sequence of mixtures. Assuming that inattention does not prevent the creation of a memory trace of the sound stimulus, attention can in principle be diverted away during the target repetition, without impairing the process by which segregation is later queried.

We used a dual task design (Figure 1) in which sound-mixture sequences were presented along with stimuli for a “decoy” task that required continuous monitoring. Attentional load was manipulated by using both a high demand (HD) condition and a control condition (low demand; LD) of each decoy task. In each case, the HD and LD conditions were based on the same stimuli and response procedure, but the LD condition was designed to require minimal attentional resources, to control for any effects of a dual task unrelated to attention. Participants were instructed to focus on the decoy task (presented to them as the focus of the experiment) and to perform the mixture tasks to the extent that they could. An online tally of overall performance was provided to subjects after each trial, and scoring was heavily weighted toward the decoy task to help ensure that the tasks would be prioritized as instructed.[Fig-anchor fig1]

To determine whether any effects of the decoy tasks were specific to segregation, we used two kinds of mixture sequences (Figure 1) that differed in whether they elicited concurrent sound segregation. We hypothesized that if sound segregation requires attention then performance should be worse under the concurrent HD decoy task than the LD decoy task, with larger effects for the segregation task (SEG) than for the control (oddball) task.

We present results from three different decoy tasks: a standard visual multiple object tracking (MOT) task, a rapid serial visual presentation (RSVP) digit encoding task (that required visual monitoring but also plausibly involved auditory working memory (WM), similar to the tasks used in Macken et al., 2003), and a demanding auditory monitoring task. The data provide converging evidence that any effect of attention on repetition-based segregation is small at best. In all cases, segregation performance remained high irrespective of a variety of objectively demanding decoy tasks. Moreover, the small effect of attention was not specific to segregation per se and appears to instead reflect a more general attentional effect on auditory processing.

## General Method

### Synthetic Sound Stimuli

**Figure 1 fig1:**
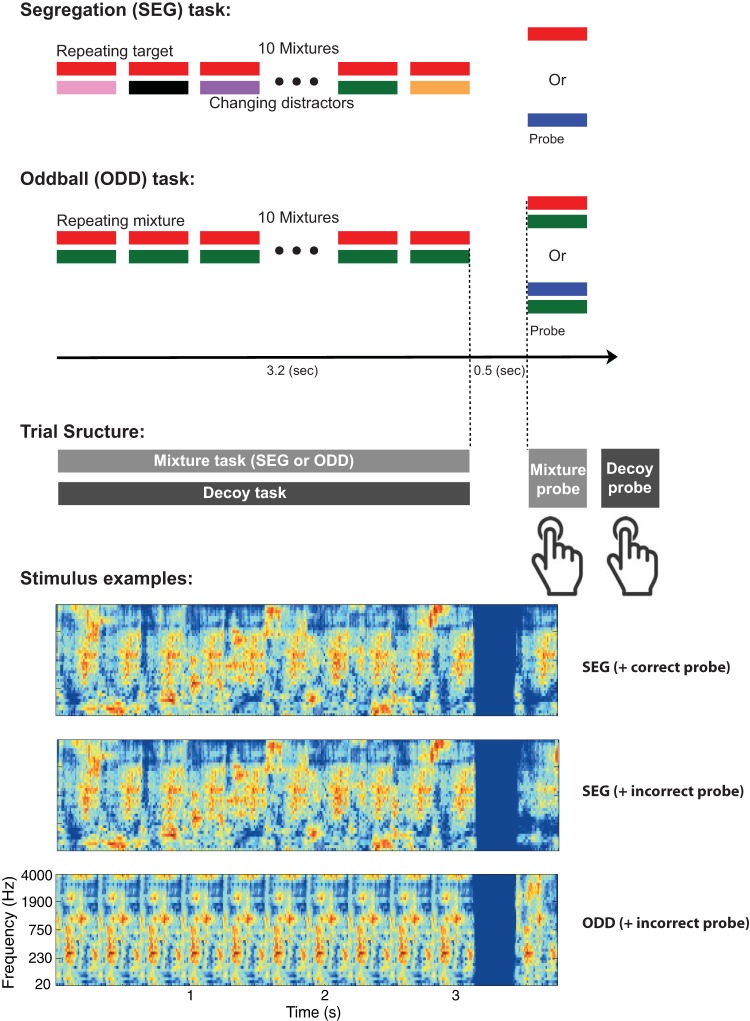
Schematic representation of the mixtures stimuli and trial structure. In the “segregation” (SEG) task, the stimulus consisted of a target sound (red (top) bars) repeatedly presented 10 times, each time with a different distractor (different colored bars). The mixture sequences were followed by a probe sound, and listeners were asked to judge whether the probe sound had been present in the mixture sequence. The probe was either the repeating target sound or a distinct sound. In the “oddball” (ODD) task, the stimulus consisted of 10 identical mixtures, followed by a probe mixture that was either identical to the repeated mixtures, or different by virtue of one of the original sounds composing the mixture having been replaced by a different sound. Cochleograms of example SEG and ODD sequences are provided at the bottom of the figure. Experimental trials were structured such that mixture sequences (SEG or ODD) were presented concurrently with decoy task stimuli to which subjects were instructed to attend. Following the presentation, subjects first responded to the mixtures probe and then to the decoy probe (separate keyboard buttons). Feedback was provided at the end of each trial. See the online article for the color version of this figure.

The stimulus generation procedure was identical to that in McDermott et al. (2011), and the reader is referred to that article for detailed information and motivation about the generative model used to construct the stimuli. In brief, 320-ms long synthetic stimuli were generated by sampling cochleograms from a multivariate normal distribution whose covariance matrix mirrored that measured in natural sounds (e.g., spoken words and animal vocalizations), and then imposing the cochleogram on a time-frequency decomposition of samples of pink noise. Cochleograms were generated by passing a signal through an auditory filterbank (with center frequencies spanning 20–4,000 Hz, equally spaced on an ERB_N_ (Equivalent Rectangular Bandwidth) scale; Glasberg & Moore, 1990) and measuring the rms level within a set of time windows (20 ms in duration, 50% overlap) applied to the resulting subbands. Cochleograms were imposed on noise by (a) generating the time-frequency decomposition of a noise signal using the same filter bank and time windows, (b) rescaling the subband content within each window to have the rms level specified in the cochleogram, (c) summing the content of each window to yield new subbands, and (d) inverting the subband transform to yield an audio signal. Matlab code for generating the experimental stimuli is available on JHM’s lab website. The resulting signals (Figure 1) shared the second-order modulation statistics of natural sounds but lacked abrupt temporal onsets and harmonic temporal structure (captured by higher-order correlations)—acoustic cues which are crucial for sound segregation (Culling & Darwin, 1993; Darwin, 1981; Darwin, 1984; de Cheveigné, Kawahara, Tsuzaki, & Aikawa, 1997; de Cheveigné, McAdams, Laroche, & Rosenberg, 1995; de Cheveigné, McAdams, & Marin, 1997).

The synthetic sounds were used in two tasks (Figure 1). In the SEG task, the stimulus consisted of a target sound repeatedly presented 10 times, each time with a different distractor (as in McDermott et al., 2011). The mixture sequences were followed by a probe sound, and listeners were asked to judge whether the probe sound had been present in the mixture sequence. The probe was either (equiprobably) the repeating target sound or a distinct sound (henceforth referred to as “incorrect probe”). In the latter case, the probe was constrained so that it was physically consistent with at least one of the presented mixtures (i.e., it never had more energy than the mixture; see more details in McDermott et al., 2011). Target, distractor, and incorrect probe sounds were all statistically comparable. Each token was presented only once to each subject. McDermott et al. (2011) demonstrated that listeners are able to determine whether the probe is identical to the repeating target with high accuracy and provided evidence that this ability reflects the segregation of the target from the background by virtue of its repetition. The experiments herein replicated the basic effect.

In the “oddball” task (hereafter abbreviated as ODD task) a sequence of 10 identical “target” mixtures was presented, followed by a probe mixture which was either identical to the target (50% of the trials) or that was different by virtue of one of the original sounds composing the mixture having been replaced by a different sound that was generated via the same procedure used for the nonmatching probes in the SEG task. The ODD task thus merely required listeners to detect an oddball sound when it occurred. The task had similar physical content to the SEG task and comparable structure but did not elicit or require any segregation processes (because single mixtures of these stimuli do not segregate), such that the task could be performed simply by comparing the spectro-temporal patterns produced by the mixtures. In pilot experiments, we found the participants could identify the oddball probe even with a single presentation of the mixture (this was also one of the conditions of Experiment 1 in McDermott et al., 2011), indicating that it did not depend on repetition-based segregation.

### Decoy Stimuli and Trial Structure

The sequences of sound mixtures were presented concurrently with a distraction task (here referred to as “decoy”), which differed for each of the reported experiments. The general trial structure is schematized in Figure 1 (center). Listeners were instructed to attend to the decoy task (which was designated to them as the main focus of the experiment). Trials began with a ∼3.2-s period (see details for each experiment) during which the decoy stimuli and mixture sequences were concurrently presented. The mixture sequence probe was presented 0.5 s after the end of the sequence. The decoy task probe was presented immediately after the listeners’ button press in response to the mixture task. Responses to both probes were to be executed within a 5-s interval—after which the program noted “no-answer” and excluded the trial from analysis. Participants practiced this rapid response procedure before the main experiment, such that “no response” trials occurred very rarely. Feedback as to the correctness of both responses was provided on the screen for 1.5 s and the following trial commenced an additional 1.5 s later. Listeners were instructed to respond as accurately as possible on the decoy task and guess as best they could on the mixtures task. To reinforce these guidelines, performance on each trial was scored such that decoy responses were worth significantly more points than mixture responses (8 and 1 points, respectively). Points obtained on each trial and the cumulative gains were displayed at the end of each trial. Participants aimed to score as many points as possible overall.

To measure the effect of attentional engagement on segregation, and to control for simple dual task demands, each experiment contained two types of “decoy” tasks: an HD task that required high concentration and placed increased load on cognitive/perceptual resources, and a LD control task based on the same stimuli but requiring minimal processing resources. All HD tasks were designed to maintain the listener’s attentional focus away from the mixture sequences and were based on monitoring rapidly presented signals, minimizing the opportunity for attentional “glimpses” toward the mixtures sequence. Tasks were adjusted, based on individual performance, to maximally tax attentional resources. Each experiment was arranged in four blocks: SEG_HD, SEG_LD, ODD_HD, ODD_LD (order counterbalanced across participants). Performance on each task was quantified by computing a *d*′ score for each participant and condition (except for the decoy task in Experiment 1 in which percentage correct was used because multiple targets were tracked on each trial).

All stimuli were generated digitally using MATLAB 7.6 (The Mathworks, Natick, MA) and Psychophysics Toolbox extensions (Brainard, 1997; Kleiner et al., 2007; Pelli, 1997) on a computer (MacBook Air, Apple Inc.). The sampling frequency was 24,000 Hz, and resolution was 16 bits. Stimuli were presented through a digital audio interface (UA-25EX, Roland Corporation, Japan) and headphones (HD555, Sennheiser Electronic Corporation, Germany), presented at a comfortable listening level of 60 to 70 dB SPL (set individually for each subject).

### Procedure

Testing was conducted in an acoustically shielded room. Before the beginning of the experiment proper, participants practiced each task (SEG, ODD, HD, LD) separately. All participants performed the ODD and LD tasks at ceiling, but SEG and HD often required some practice. It was important to make sure that baseline performance in SEG was sufficiently high in order for potential effects of attention to be resolvable. Good performance on HD was also necessary, so as to confirm that subjects were indeed engaged by the decoy task. After repeating eight blocks (20 trials each) of each task, only participants who reached *d*′ > 1 in SEG and *d*′ > 2.5 in HD continued to the main experiment. About 30% of those initially screened were excused from the study because of failure to reach the threshold in the SEG task. The practice session concluded with four short blocks in which participants practiced the dual-task combinations.

### Participants

All participants reported normal hearing and had no history of neurological disorders. They were paid for their participation. The experimental procedures were approved by the Research Ethics Committee of University College London, and written informed consent was obtained from each participant.

## Experiment 1: Distraction by a Visual-Based Decoy Task

This experiment used an MOT task (Pylyshyn & Storm, 1988), in which participants are required to visually track the position of a subset of moving dots on a computer screen. The task is commonly used to study visual attention and WM and is known to be attentionally demanding (Alvarez & Franconeri, 2007; Doran & Hoffman, 2010; Drew, McCollough, Horowitz, & Vogel, 2009; Rouhinen, Panula, Palva, & Palva, 2013; Tombu & Seiffert, 2008). Because of its hypothesized reliance on the attentional mechanisms commonly employed by observers to track relevant objects in natural environments, for example, while driving (Lochner & Trick, 2014), the MOT task is arguably an ecologically relevant means with which the availability of central attentional resources can be systematically manipulated. The main parameters that affect performance limits in this task are the number of objects in the display, how they are spaced, and the speed with which they move (Bettencourt & Somers, 2009; Culham, Cavanagh, & Kanwisher, 2001; Pylyshyn & Storm, 1988). The latter was manipulated in the present experiment.

**Figure 2 fig2:**
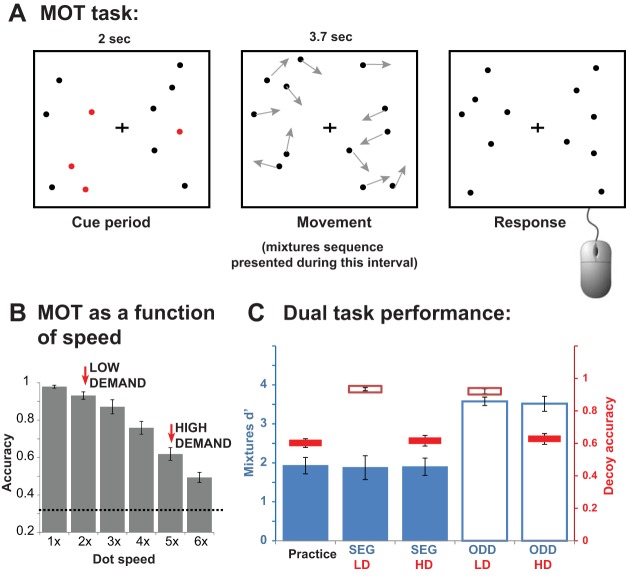
Stimuli and results for Experiment 1: Multiple Object Tracking (MOT) decoy task. (A) MOT task structure: At the start of a trial (2-s cue period), 12 static dots (eight black; four cued in red) appeared on the computer screen. At the start of the tracking period (3.7-s in duration) the color of the cued dots change to black and all dots began moving. At the end of the tracking period all dots stopped abruptly. Participants first responded to the auditory probe (see trial structure in Figure 1), and were then prompted to click on the MOT targets. (B) MOT performance as a function of dot speed (results of pilot experiment). Chance level is indicated with a dashed line. The 2× condition was chosen for the low demand (LD) task, and the 5× condition was chosen for the high demand (HD) task. (C) Results of the dual task experiment for each combination of mixtures and decoy task. Performance on the decoy task in plotted in red (horizontal bars; solid segments for HD; outline segments for LD; relative to the axis on the right) and performance on the mixtures task is plotted in blue (solid bars for the segregation [SEG] task; outline bars for the oddball “ODD” task; relative to the axis on the left). The practice data are single-task performance on the SEG condition and HD condition prior to the beginning of the experiment proper. Error bars here and elsewhere plot *SEM*s. See the online article for the color version of this figure.

We used displays in which participants were required to track 4 out of 12 moving dots. Based on previous evidence that increasing the speed of the dots in the display increases the attentional demands of tracking (Alvarez & Franconeri, 2007; Feria, 2013; Tombu & Seiffert, 2008;), movement speed was adjusted to produce two levels of difficulty (Figure 2B). In one condition (LD), the dots moved slowly and tracking performance was close to ceiling. In the other condition (HD), the dots moved 2.5 times faster, and tracking became significantly more demanding (though still performed with a high success rate).[Fig-anchor fig2]

Under the hypothesis that MOT draws on central, flexibly deployable, attentional resources (Alvarez & Franconeri, 2007; Bettencourt & Somers, 2009; Tombu & Seiffert, 2008), increasing the speed of the dots should deplete resources available for other perceptual processes. Therefore, if auditory segregation requires (central) attention, performance in the SEG task under HD should be reduced relative to that under the LD condition.

### Method

The visual stimuli were generated with the same procedure used by Franconeri et al. (see: http://viscog.psych.northwestern.edu/projects/FJSInPrep.html). Figure 2A schematizes the MOT task structure. At the start of a trial (2-s cue period), 12 static dots (eight black; four cued in red) appeared on the computer screen. They then began moving, and the color of the cued dots changed to black, initiating the 3.7-s tracking period (the 3.2-s auditory mixture sequences were presented from 0.5 s into this interval). Objects moved in pseudo random paths as described in http://viscog.psych.northwestern.edu/projects/FJSInPrep.html (second set of experiments). At the end of that period, all objects stopped abruptly. Participants first responded to the auditory probe, as described previously, and then were prompted to click on the MOT targets. Accuracy was calculated by tallying the number of correctly identified targets (100% = all identified; 75% = 3 out of 4 identified correctly, etc.).

Speed was manipulated by sampling a continuous video of these moving objects and displaying either every animation frame (speed = 1), every other frame (speed = 2), and so forth. We conducted a pilot experiment to quantify performance as a function of dot speed. Each condition (speed = 1, 2, 3, 4, 5, and 6) was presented in a separate block (20 trials), with block order randomized across participants. The results are shown in Figure 2B. It was important that the HD task be difficult (i.e., taxed attentional resources) but not impossible, so as to continually engage the subjects. The speed = 5 condition was chosen for this purpose. The speed = 2 condition was chosen for the LD task such that overall the HD condition was 2.5× faster than the LD condition.

The main experiment lasted approximately 120 min. Participants were presented with two blocks for each stimulus combination (SEG_HD, SEG_LD, ODD_HD, ODD_LD); order counterbalanced across participants. A single block consisted of 40 trials and took around 8 min to complete.

### Participants

Ten participants (mean age = 28.5 years; two females) took part in the pilot experiment. Ten participants (different from those in the pilot; mean age = 29.2 years; two females) participated in the main experiment.

### Results and Discussion

#### Decoy tasks

Accuracy was approximately 62% for HD and 93% for the LD task. These levels are almost identical to those measured in the pilot experiment, under single task conditions (Figure 2B). A repeated-measures analysis of variance (ANOVA) with mixtures task type and decoy task type as factors showed only a significant main effect of decoy task type, *F*(1, 9) = 160.8, *p* < .001, and no interactions, indicating that the task was successful at manipulating load. The absence of an effect of the mixtures task—indicating that MOT performance did not vary as a function of the auditory task—suggests that participants followed the instructions to prioritize the visual task.

#### Auditory mixture tasks

A repeated-measures ANOVA with mixture task type and decoy task type as factors showed a significant main effect of mixture task type, *F*(1, 9) = 59.3, *p* < .001, with no main effect of the decoy task type (*p* = .89) and no interaction between the two factors (*p* = .68). As expected, performance on the ODD task (*d*′ about 3.5) was better than that on SEG (*d*′ about 1.9). However, neither task was affected by the load in the decoy task. In fact, the performance on SEG under HD was identical to the performance level measured during the practice session when the SEG sequences were presented in isolation, without a competing visual task (repeated-measure ANOVA over data from practice, SEG_HD and SEG_LD: *F*(1, 9) = 0.05, *p* = .95).

The results indicate that a concurrent MOT task had no effect on listeners’ ability to detect the repeating auditory targets, despite its intense sustained demands on central attentional resources. These results are consistent with previous reports of a lack of interference between a primary MOT task and a secondary auditory task (Arrighi, Lunardi, & Burr, 2011; Sculthorpe et al., 2008; though see Tombu & Seiffert, 2008) albeit with relatively simple auditory tasks (frequency discrimination and odd-ball detection), comparable to the ODD task used here.

## Experiment 2: Distraction by an RSVP Memory Load Task

**Figure 3 fig3:**
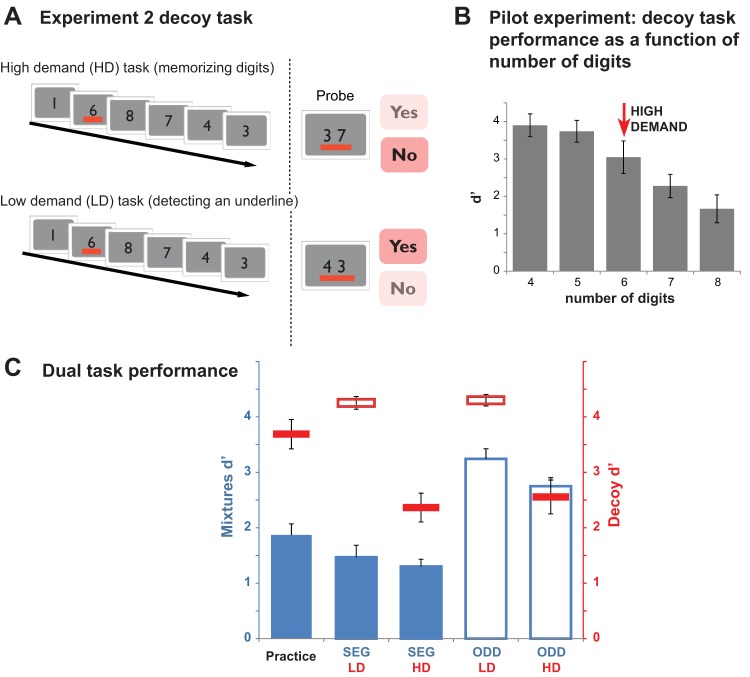
Stimuli and results for Experiment 2A: Rapid Serial Visual Presentation (RSVP) memory load decoy task. (A) RSVP task structure: Stimuli consisted of a rapid serial presentation of single digits (from 1 to 8) at the center of the computer screen. The digit series were composed of five or six digits, depending on each participant’s performance in the practice session. In half of the trials, one of the digits (randomly chosen) was underlined in red. In the high demand (HD) task, participants were required to memorize the sequence of digits (and to ignore the underline if it was presented). At the end of the trial (following the response to the mixtures probe), a pair of digits (probe) was presented on the screen. These were always digits that appeared in the preceding sequence and participants were required to report (by pressing a “Yes” or “No” button) whether the two digits were exactly consecutive in the preceding sequence (in the example shown the correct answer is “No”). In the low demand (LD) task, participants were required to report whether an underline appeared within the sequence (in the example shown the correct answer is “Yes”). The probe structure (two digits and an underline) was identical in the HD and LD tasks however, participants were instructed to ignore the content of the probe in the LD tasks and respond regarding the presence of the underline only. (B) RSVP performance as a function of number of digits (results of pilot experiment). The six-digits condition was chosen for the main experiment (although some participants who performed poorly with six digits ran the main experiment on the five-digits condition). (C) Results of the dual task experiment for each combination of mixtures and decoy task. Conventions are as in Figure 2. See the online article for the color version of this figure.

Experiment 2 used a decoy task based on rapid serial visual presentation (RSVP) of digits that subjects were instructed to memorize and subsequently report (Figure 3A). The task is similar to that used in experiments on the “irrelevant sound effect” (see Macken et al., 2003) except that the rate at which the digits were presented was substantially faster in the present experiment. The rapid stimulation required participants to maintain continuous attention with minimal opportunity to shift attention to the auditory mixture sequence.[Fig-anchor fig3]

The decoy stimuli were presented visually, and so did not interact with the mixture stimuli at the sensory level. However, the task explicitly drew on phonological loop WM resources (Baddeley, 1986; Buchsbaum & D’Esposito, 2008), which could compete with auditory processing more centrally. In general, accumulating evidence suggests that WM tasks at least partly engage domain general systems, limiting the availability of resources to other stimulus streams (Carlyon et al., 2003; Dalton, Santangelo, & Spence, 2009; Ghatan et al., 1998; Haroush, Deouell, & Hochstein, 2011; Klemen, Büchel, & Rose, 2009; Rissman, Gazzaley, & D’Esposito, 2009).

The difficulty of the RSVP (HD) task was adjusted according to each participant’s individual abilities so as to maximally draw on available processing resources. The control (LD) task was based on exactly the same stimuli and response requirements but involved simple “pop-out” detection (red line under one of the digits). If segregation depends on the availability of central attentional resources, a HD concurrent task should result in diminished SEG performance (relative to the corresponding LD task).

### Experiment 2A

#### Method

Figure 3A provides a schematic representation of the decoy task used in Experiment 2. The decoy stimulus consisted of a rapid serial presentation of single digits (from 1 to 8) at the center of the computer screen (digit size was approximately 7 cm by 5 cm at a viewing distance of 50 cm). The total duration of the presentation was 3.2 s (same duration as the mixture sequences). The digit sequence was composed of five or six digits, depending on each participant’s performance in the practice session (see below). Each digit was presented for 0.53 s in the six-digit condition and 0.62 s in the five-digit condition. In 50% of the trials, one of the digits (randomly chosen) was underlined in red. Participants were instructed to fixate at the center of the screen (a fixation cross was provided between trials). In the HD task, they were required to memorize the sequence of digits (and to ignore the underline if it was presented). At the end of the trial (following the response to the mixtures probe), a pair of digits (probe) was presented on the screen. These were always digits that appeared in the preceding sequence and participants were required to report (by pressing a “Yes” or “No” button) whether the two digits were exactly consecutive (e.g., in the example shown in Figure 3A, the correct answer is “No”). In the LD task, participants were required to report whether an underline appeared within the sequence (symbolized by the red underline beneath the probe digits).

To ensure that the HD task was sufficiently demanding, we conducted a pilot experiment to determine how many digits could be memorized within a 3.2-s interval. Each condition (4, 5, 6, 7, and 8 digits) was presented in a separate block, with block order randomized across participants. Two blocks of 20 trials each were administered for each condition. The results are shown in Figure 3B. It was decided to set the number of digits to six because performance began to drop off at that number, suggesting that six digits was about the average capacity across subjects.

Standard paradigms for measuring effects of WM load usually present the to-be-remembered token (e.g., digit) sequence followed by additional stimuli (whose processing might interact with the memory load) during the retention interval (e.g., Dalton et al., 2009; de Fockert, Rees, Frith, & Lavie, 2001). In contrast, here the digits were presented sequentially along with the unfolding auditory mixture sequence (see also Macken et al., 2003). The successive presentation aspect of the task was vital for continuously drawing attention away from the acoustic stimuli, but had the consequence that memory load in the HD conditions grew over the duration of the trial. We note, however, that the demands of the HD task, including the need to recognize and encode rapidly presented information, were arguably greater from the outset than those for the LD task, which was based on visual pop-out (a red line among a sequence of black stimuli).

As in Experiment 1, participants practiced all the tasks separately before beginning the experiment. Participants who did not reach *d*′ above 2.5 in the HD task with six digits, practiced the same task with five digits. After 40 additional trials those participants who obtained *d*′ above 2.5 ran the main experiment with five digits; others were discharged.

The main Experiment lasted approximately 120 min. Participants were presented with two blocks for each stimulus combination (SEG_HD, SEG_LD, ODD_HD, ODD_LD). A single block consisted of 40 trials and took around 8 min to finish. Subjects were allowed short breaks between blocks.

#### Participants

Eight participants (mean age = 26.3 years; six females) took part in the pilot experiment.

Seventeen participants were tested in Experiment 2A. Six were excluded because their performance on the visual decoy task indicated an effect of the auditory task (a *d*′ difference of more than 1 between SEG_LD and ODD_LD, or SEG_HD and ODD_HD), implying that they had not prioritized the decoy task as per the instructions. This experiment was conducted first chronologically and the experimenter was still perfecting the instructions regarding the decoy task prioritization. The issue did not occur in subsequent experiments. The presented data are therefore from the remaining 11 participants (mean age = 28.8 years; five female). Four of the participants performed the decoy task with five digits.

#### Results and discussion

##### Decoy tasks

Performance on the decoy task is indicated with red lines on Figure 3C. A repeated-measures ANOVA with decoy task and mixtures task as factors showed a significant main effect of decoy task, *F*(1, 16) = 43.1, *p* < .001, and no interactions, indicating that the task was successful at manipulating load.

The sizable difference in HD performance between the practice and the dual task setting (paired sample *t* test: *t* = 4.54, *p* = .001), reflects the added demands of the dual task conditions: In addition to potential interference during the concurrent presentation of the two stimulus streams, participants had to maintain the digit sequence in WM while responding to the mixtures probe. This produced a decrement in HD performance irrespective of the mixture task being performed.

##### Auditory mixture tasks

Performance on the mixtures task is indicated with blue bars on Figure 3C. A repeated-measures ANOVA with mixture task and decoy task as factors confirmed a significant main effect of mixture task, *F*(1, 10) = 78.64, *p* < .001, and decoy task type, *F*(1, 10) = 9.9, *p* = .01, with no interaction between the two factors (*p* = .22). The results indicate a small, but consistent, decline in performance for both the SEG and ODD tasks during the HD, relative to the LD decoy tasks. Similarly, there is a significant, but relatively small, decline in SEG performance between the practice and dual task setting) paired sample *t* test: *t* = 3.02, *p* = .013). The results overall demonstrate that SEG performance is only slightly affected by a concurrent memory loading RSVP task.

### Experiment 2B: Time-Reversed Probes

Experiments 1 and 2A used incorrect probes which by virtue of being conditional samples from a random process typically had long-term power spectra that were distinct from that of the targets. As a result, the task could in principle have been performed by comparing the probe to a memory trace of the long-term spectra of the mixture sequence. Although the original experiments of McDermott et al. (2011) controlled for this possibility, it seemed plausible that inattention might force listeners to use a simpler strategy than they would use when directing attention to the mixture sequence as in McDermott et al.’s experiments. In Experiment 2B, we thus incorporated the control experiment from McDermott et al., in which the signals used for the incorrect probes were a time-reversed version of the target. The probes and targets thus shared the same long-term spectral structure and only differed in their temporal properties.

#### Method

The general procedure was mostly identical to that in Experiment 2A, except that two types of incorrect probe were used: A standard incorrect probe (ST; as in Experiments 1 and 2A) and a time-reversed incorrect probe (TR), which was generated by time-reversing the target (for this condition, we used target sounds that were asymmetric in time; for more details see Experiment 3b in McDermott et al., 2011). Both conditions were randomized within the same block (including in the practice session), thus doubling the number of trials per condition. The total number of trials was 320 in each condition (SEG_HD, SEG_LD, ODD_HD, ODD_LD) consisting of 80 ST incorrect probes, 80 TR incorrect probes, and 160 targets. Participants were not informed about the stimulus differences and received the same instructions as in Experiment 2A. The experiment lasted approximately 180 min.

#### Participants

Twelve new participants (mean age = 27.75 years; four females) participated in the main experiment. Two of the participants performed the decoy task with five digits.

#### Results and discussion

**Figure 4 fig4:**
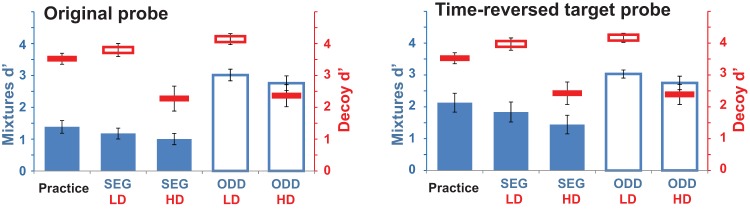
Results of Experiment 2B, with time-reversed probes on half of the trials. Conventions are as in Figure 2. See the online article for the color version of this figure.

The results are plotted separately for the standard probes and the time-reversed probes (Figure 4). It is apparent that the results with standard probes replicate those of Experiment 2A, and that the pattern of results is qualitatively similar for time-reversed probes, with a small effect of decoy task for both probe types.[Fig-anchor fig4]

##### Decoy tasks

A repeated-measures ANOVA on decoy task performance with decoy task type, probe type, and mixtures task type as factors again showed only a significant main effect of decoy task type, *F*(1, 11) = 31.0, *p* < .001, and no interactions.

##### Auditory mixture tasks

A repeated-measures ANOVA with probe type, mixture task type, and decoy task type as factors showed significant main effects of mixture task type, *F*(1, 11) = 109.4, *p* < .0001, and decoy task type, *F*(1, 11) = 8.7, *p* = .013, as before. Thus, the time-reversed probes did not enhance the effect of the decoy task on the mixture tasks.

The time-reversed and standard probes were not matched for difficulty, and the reader may notice that performance in the SEG task was slightly better for time-reversed than standard probes (Figure 4). This difference was statistically significant, producing an overall effect of probe type, *F*(1, 11) = 20.1, *p* = .001, and an interaction between probe type and mixtures task type, *F*(1, 11) = 6.7, *p* = .026. Overall, however, these results replicate the performance in Experiment 2A, by demonstrating that the ability to recover the spectro-temporal structure of a repeating target was only slightly affected by a competing, HD, RSVP task.

## Experiment 3: Distraction by an Auditory-Based Counting Task

The decoy task in Experiment 3 involved auditory stimuli, and so utilized a dichotic design both to minimize masking and other forms of peripheral interference between the stimuli for the two tasks, and to facilitate the diversion of attention from the mixture sequence. The mixture sequences (identical to those in the previous experiments) were presented to one of the ears. The other ear received a rapid (∼4 Hz) sequence of tone-pips. In the HD task, participants were instructed to count the tones. In the LD task, participants detected a salient frequency deviant present in half of the trials. As in Experiment 2, the difficulty of the HD task was set according to the capacity limits of each listener to assure maximal depletion of relevant processing resources.

The task was designed such that the decoy and mixture stimuli would not interfere at the periphery. However, a large body of work suggests that concurrently presented signals vie for processing resources such that directed attention to one of the streams often results in reduced brain response to the other (Alho et al., 1999; Bidet-Caulet et al., 2007; Bidet-Caulet, Mikyska, & Knight, 2010; Chait et al., 2012; Woldorff, Hillyard, Gallen, Hampson, & Bloom, 1998). We therefore expected that this decoy task would markedly impair performance on the mixtures task if the latter requires the availability of general auditory processing resources.

### Method

**Figure 5 fig5:**
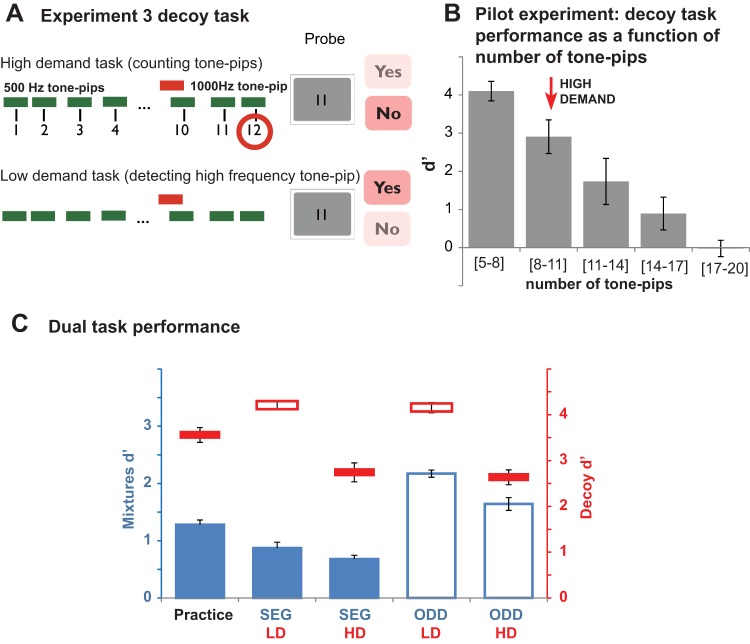
Stimuli and results for Experiment 3: Auditory counting decoy task. (A) Counting task structure: Trials consisted of a 3.2-s long sequence of a variable number of identical, 100-ms tone pips (green bars). In half of the trials, a high frequency tone pip (“deviant”; red bar) was presented at a random time during the trial. After the sequence (following the probe for the mixtures sequence), a cue, in the form of a number, was presented on the screen. That number was either identical to the number of tone-pips in the sequence (excluding the deviant, if present) or differed from it by 1. In the high demand (HD) task, participants were required to count the tones (ignoring the high frequency deviant) and determine (by pressing “Yes” or “No”), whether the number in the probe matched that total (in the example, the correct answer is “No” because 12 tones were presented). In the low demand (LD) task, participants ignored the content of the probe and reported whether a high-frequency tone was present in the sequence (in the example, the correct answer is “Yes”). (B) Counting performance as a function of number of tone pips (results of pilot experiment). The [8–11] tone-pips condition was chosen for the main experiment (although some participants who performed at ceiling ran the main experiment on the [11–14] condition). (C) Results of the dual task experiment for each combination of mixtures and decoy task. Conventions are as in Figure 2. See the online article for the color version of this figure.

The mixture stimuli were identical to those used in the previous experiments, except that they were now delivered to only one of the ears (including during the single-task practice session) while the other ear received the decoy task stimuli. Figure 5A is a schematic representation of the decoy task in Experiment 3. Trials consisted of a 3.2-s long sequence of a variable number of 500 Hz tone pips, each 100 ms long and presented at 71 dB SPL. Intertone intervals (ITI) varied randomly (min ITI was 0.2 s; max ITI depended on the number of tones in the sequence, as described below). A high frequency (1,000 Hz), 100-ms tone-pip (“deviant”) was presented concurrently with the sequence in 50% of the trials. The timing of the deviant’s appearance was determined randomly on each trial. In the HD task, participants were required to count the 500 Hz tones (ignoring the high frequency deviant) and determine (by pressing “Yes” or “No”), whether the number in the subsequent probe matched their tally. After the sequence, following the probe for the mixture sequence, a tally probe appeared on the screen. That number was either identical to the number of 500 Hz tone pips in the sequence or differed from the number of presented tone pips by 1 (this made it difficult to guess the correct answer). In the LD task, participants ignored the content of the probe and just reported whether a high frequency tone was present in the sequence. Ear conditions were counterbalanced across blocks. The other procedures were identical to those in preceding experiments.[Fig-anchor fig5]

To set the number of tones in the sequence, we conducted a pilot experiment in which we tested participants’ ability to maintain a tally of an increasing number of tone-pips. Sequences consisting of [5–8], [8–11], [11–14], [14–17], or [17–20] tone pips were presented in separate blocks (32 trials each), with block order randomized across participants. The results are shown in Figure 5B. The number of tone-pips was initially set to [8–11] tones. Participants who reached *d*′ > 2.5 after 40 practice trials moved on to try the more difficult [11–14] tones condition.

### Participants

Ten subjects (mean age = 28.4 years; six females) participated in the pilot experiment. Seventeen subjects (mean age 26.7 years; eight females) participated in the main experiment. Six participants performed the decoy task with [8–11] tones. The decoy task for the remaining participants was set to [11–14] tones.

### Results and Discussion

#### Decoy tasks

Performance on the decoy tasks is indicated with red lines on Figure 5C. A repeated-measures ANOVA with decoy task type and mixtures task type as factors demonstrated a significant main effect of decoy task type, *F*(1, 16) = 70.7, *p* < .001 only, indicating that the task was successful at manipulating load.

The reduction in performance between the practice and the dual task setting (paired sample *t* test: *t* = 3.8, *p* = .001) is plausibly because of some interference between the two concurrent stimulus sequences. Overall, however, the high performance levels in the HD task, and the lack of effect of mixture-task type on decoy task performance are indicative of sustained focused attention onto the tone-pip stream.

#### Auditory mixture tasks

Performance on the SEG and ODD mixture tasks is indicated by blue bars on Figure 5C. The increased demands of the main experiment are apparent in the reduction in SEG performance between the practice and dual task setting (for both LD and HD; paired sample *t* test: *t* = 3.2, *p* = .005). We note also that overall SEG performance in this experiment was the lowest of the three experiments reported. This may be because of a combination of individual differences and the fact that the sounds were presented to only one ear, reducing the overall loudness of the stimuli. Informal listening by the authors suggested that the SEG task indeed became harder when the signals were presented monaurally.

As in Experiment 2, the effect of attention was significant, but small, and similar across SEG and ODD tasks. A repeated-measures ANOVA with mixtures task type and decoy task type as factors confirmed a significant main effect of the mixtures task, *F*(1, 16) = 97.0, *p* < .001, as well as the decoy task, *F*(1, 16) = 33.6, *p* < .001, with no interactions. It is important that while SEG performance was reduced somewhat during the concurrent decoy task it remained significantly above floor (one-sample *t* test: *t* = 10.3, *p* < .001). Listeners were thus able to detect the repeating spectro-temporal patterns in the ignored ear despite the fact that they were engaged by tracking the number of rapidly presented tone-pips. The SEG and ODD task results did not change (in terms of statistical significance—*p* < .001 in all cases—or the size of the effect) when analyzing data from only the top 50% of the performers on the HD task or those subjects who performed best on the SEG task during practice.

### Discussion

We investigated the effect of attentional load on an auditory segregation task that required listeners to recover the structure of repeating “targets” in a background of distractors. Attention was manipulated by asking subjects to concurrently perform high- or low-demand versions of three “decoy” tasks. The HD decoy tasks were designed to entail continuous monitoring, thereby drawing attention away from the sound mixture stimuli. Matched control decoy tasks, in contrast, allowed for ample capacity to be allocated to the mixture sequence. Our main finding is that performance was only slightly affected by exhaustion of attentional resources, and that this effect was not specific to segregation per se. The small reduction in performance is consistent with theories of limited perceptual capacity (e.g., Cartwright-Finch & Lavie, 2007; Lavie, 2005; Lavie, Beck., & Konstantinou, 2014). However, because similar-sized attentional effects were observed for the oddball control task (ODD) that did not require any segregation, it seems that the effect reflects a general attentional influence on auditory processing.

#### Dual-task limitations and caveats

The dual task design that we employed required participants to make judgments about both the decoy and the mixture stimuli. The advantage of this approach is that attentional load can be quantified and manipulated so as to maximally (HD tasks) or minimally (LD tasks) deplete available resources, the effect of which can be measured on the task of interest. The weakness of the approach is that subjects are inevitably incentivized to monitor the stimuli from which attention is intended to be diverted. We sought to minimize this incentive by prioritizing the decoy task in the instructions (which described it as the main focus of the study) and scoring (higher weighting for the decoy task), of which subjects were reminded when feedback was given after each trial. But can we ensure that attention was in fact diverted from the mixture stimuli?

If the mixture tasks were drawing attention away from the decoy task, one might expect decoy performance to vary as a function of mixture task (SEG vs. ODD). This is because the ODD task was intended to be less demanding than the SEG task (with substantially higher performance in ODD than SEG), such that if subjects diverted attention toward the mixture streams, they might do so to a greater extent in the SEG task than in the ODD task, producing lower performance in the decoy task. Consistent with the idea that subjects on the whole were successfully prioritizing the decoy task, this trend was observed only for a few subjects (see Experiment 2), who were excluded from analysis.

Another indication of incomplete inattention could be if the decoy task performance declined from the practice block (where it was performed in isolation) to the experiment proper (where it was performed concurrently with the SEG and ODD tasks). Such a decrease was indeed observed in Experiments 2 and 3, but not in Experiment 1. Because the decoy tasks in Experiments 2 and 3 involved auditory echoic memory and an auditory stimulus, respectively, whereas that of Experiment 1 was exclusively visual, it seems most parsimonious to suppose that the effect on the decoy task reflects some sort of sensory interference rather than diverted attention per se. However, even if some attention was allocated to the mixture sequences in Experiments 2 and 3, it remains the case that performance on the (demanding) decoy tasks was high overall when performed concurrently with the mixture tasks (*d*′ > 2), that performance on the SEG and ODD tasks was only modestly affected by a very pronounced manipulation of concurrent load, and that this effect was indistinguishable for the SEG and ODD tasks. Thus, irrespective of whether some attention was directed to the mixture stimuli, our results indicate that segregation by repetition is relatively robust to inattention.

#### Origins of attentional effect

Our results do not speak directly to the origins of the small but general effect of attention that we observed. The attentional effect on the SEG and ODD tasks could plausibly occur at early processing stages (e.g., Johnson & Zatorre, 2005; Kastner & Ungerleider, 2000), consistent with brain imaging evidence that early auditory onset responses are modulated by the demands of a concurrent visual task but that later processes, such as the MMN, are not affected (e.g., Dyson et al., 2005). Alternatively, it could reflect effects of attention on auditory memory, which was necessary to compare the probe sound to the target sound in the preceding mixture sequence for both SEG and ODD tasks. Overall, however, the small effect of load suggests that the memory trace for the target was established despite diverted attention. Although the ODD task could in principle have been performed by comparing the probe sound to a trace of the final repetition of the mixture, this would not have been viable for the SEG task, in which the target cannot be estimated from a single mixture (McDermott et al., 2011). Repetition-based segregation is inherently dependent on a memory buffer that develops and maintains an estimate of the repeating structure across repetitions. The results thus indicate that the relevant memory processes were largely intact throughout much of the mixture sequence despite the concurrent tasks. It seems that listeners could then access the resulting memory trace for comparison with the subsequent probe, perhaps by directing attention to the memory trace once the task stimuli had finished.

We separately examined the effects of decoy tasks that were either entirely visual (MOT) or entirely auditory (contralateral beep counting) because a priori it seemed plausible that attentional resources might be at least partially modality-specific (Chait et al., 2012; Dyson et al., 2005; Muller-Gass et al., 2006; Rees, Frith, & Lavie, 2001; Restuccia, Restuccia, Della Marca, Marra, Rubino, & Valeriani, 2005; Sculthorpe et al., 2008; Talsma, Doty, Strowd, & Woldorff, 2006; but see Raveh & Lavie, 2015). Although Experiment 1 (MOT) was the only experiment in which there was no significant effect of load, a one-way ANOVA over the difference between SEG_HD and SEG_LD across experiments was not significant (*p* = .267). There were thus not obvious qualitative differences between the effects of occupying attention with competing tasks in vision versus audition.

#### Relation to other types of sound segregation

In part because of the challenges of querying listeners about unattended stimuli, there have been relatively few investigations of the consequences of attentional load on sound segregation (reviewed in Macken et al., 2003; Murphy et al., 2013). As discussed in the introduction, the conclusions of studies using the popular A-B-A streaming stimulus have been equivocal (Carlyon et al., 2001; Carlyon et al., 2003; Gutschalk et al., 2015; Macken et al., 2003, Snyder et al., 2006; Thompson et al., 2011). Moreover, the A-B-A stimulus, although commonly studied, is arguably a questionable model for natural sound mixtures, because the mixture components are both clearly distinct and spectrally separated, and exhibit a precise, regular temporal relationship. These features give rise to idiosyncratic perceptual effects such as a slow build-up and bistability (Andreou et al., 2011; Denham & Winkler, 2006) that are not obviously shared with typical natural sound mixtures, and the attentional effects observed previously could be specific to these idiosyncrasies (see also Gutschalk et al., 2015).

Another more recently studied streaming paradigm consists of a repeated target tone stream in a background of desynchronized tone pips of varying frequency (exhibiting “informational masking,” or IM; Akram, Englitz, Elhilali, Simon, & Shamma, 2014; Elhilali, Xiang, Shamma, & Simon, 2009; Gutschalk, Micheyl, & Oxenham, 2008; Kidd, Mason, Deliwla, Woods, & Colburn, 1994). Brain responses that correlate with IM target-stream awareness are abolished when attention is diverted away from the IM stimulus, a finding taken to indicate that segregation is reliant on attention (e.g., Gutschalk et al., 2008). However, an alternative explanation is that attention affects awareness of segregation rather than segregation itself.

Compared with previous studies, our paradigm has the advantage of querying a consequence of streaming (the ability of the listener to compare a subsequent probe sound to a memory trace of a target source that was embedded in a mixture) rather than the streaming percept itself (as in the A-B-A paradigm). It also has the benefit of utilizing complex spectrotemporal ‘sources’ that overlap in much the same way that natural sounds do, unlike, for instance, typical IM stimuli that incorporate a spectral “protective region” between the target and distractors. However, it remains unclear to what extent our findings of repetition-based segregation under diverted attention will generalize to segregation by other means.

The problem of separating a sound mixture into representations of individual sources can only be solved with prior constraints on the structure of the sources. These constraints can take many forms, and could be applied by a range of processes, plausibly operating at different stages of the auditory system (Bregman, 1994; Carlyon, 2004; McDermott, 2009). Some such processes are known to utilize relatively generic statistical regularities of natural sounds, such as harmonicity, common onset, similarity over time (as in classic A-B-A streaming), and the repetition utilized in the present paper. However, listeners are also able to segregate using memories of particular familiar sounds—for example when hearing out a familiar melody interleaved with other tones, or when utilizing knowledge of language to segregate speech (Bey & McAdams, 2002; Billig, Davis, Deeks, Monstrey, & Carlyon, 2013; Bregman, 1994; Devergie, Grimault, Tillmann, & Berthommier, 2010). The influence of attention on segregation could thus plausibly depend on what is driving segregation. Our results support the hypothesis that segregation by generic statistical regularities could be largely automatic and independent of attentional resources. The role of attention in other segregation processes, such as those rooted in musical or linguistic knowledge, remains unclear, and further work will be required to determine whether they are as robust to inattention as the repetition-based segregation studied here.
